# CST: Complex Sparse Transformer for Low-SNR Speech Enhancement

**DOI:** 10.3390/s23052376

**Published:** 2023-02-21

**Authors:** Kaijun Tan, Wenyu Mao, Xiaozhou Guo, Huaxiang Lu, Chi Zhang, Zhanzhong Cao, Xingang Wang

**Affiliations:** 1 Institute of Semiconductors, Chinese Academy of Sciences, Beijing 100083, China; 2 University of Chinese Academy of Sciences, Beijing 100089, China; 3 Chinese Association of Artificial Intelligence, Beijing 100876, China; 4 Materials and Optoelectronics Research Center, University of Chinese Academy of Sciences, Beijing 100083, China; 5 College of Microelectronics, University of Chinese Academy of Sciences, Beijing 100083, China; 6 Semiconductor Neural Network Intelligent Perception and Computing Technology Beijing Key Laboratory, Beijing 100083, China; 7 Nanjing Research Institute of Information Technology, Nanjing 210009, China

**Keywords:** speech enhancement, attention mechanisms, UNET architecture, transformer

## Abstract

Speech enhancement tasks for audio with a low SNR are challenging. Existing speech enhancement methods are mainly designed for high SNR audio, and they usually use RNNs to model audio sequence features, which causes the model to be unable to learn long-distance dependencies, thus limiting its performance in low-SNR speech enhancement tasks. We design a complex transformer module with sparse attention to overcome this problem. Different from the traditional transformer model, this model is extended to effectively model complex domain sequences, using the sparse attention mask balance model’s attention to long-distance and nearby relations, introducing the pre-layer positional embedding module to enhance the model’s perception of position information, adding the channel attention module to enable the model to dynamically adjust the weight distribution between channels according to the input audio. The experimental results show that, in the low-SNR speech enhancement tests, our models have noticeable performance improvements in speech quality and intelligibility, respectively.

## 1. Introduction

Communication through voice is the most natural and efficient way [1]; however, noise often affects voice quality, damages hearing, and reduces intelligibility. The speech enhancement algorithm is a crucial way to solve this problem. It can improve speech quality and intelligibility by removing noise in speech audio. Currently, most of the existing speech enhancement algorithms are designed for speech audios with a high Signal-to-Noise Ratio (SNR) and have achieved relatively ideal performance in most scenarios with less noise [2]. However, the performance of existing algorithms is limited in scenes with high noise interference and lower SNR, such as forging factories, racing cars, extreme sports, and even battlefields. Speech enhancement tasks for low SNR scenarios are challenging [3], and related research is still in its infancy. In this paper, we propose a model for low-SNR scenarios.

With the development of deep learning technology, great progress has been made in different fields, such as computer vision(CV), natural language processing (NLP), and audio processing. In the field of speech enhancement, Lu [4] proposed a speech enhancement method based on a deep neural network in 2013, and, since then, deep learning-based methods have become mainstream. Among many network architectures, the UNET [5] architecture is widely used in speech enhancement tasks. It was originally used in image segmentation, which requires neural networks to perform semantic classification of images pixel by pixel, which shares similarities with speech separation and speech enhancement. Stoller et al. [6] use the UNET to achieve speech separation, Macartney et al. [7] use the UNET-based network to achieve speech enhancement on the audio time–domain signal and achieved state-of-the-art (SOTA) at that time. Hao et al. [8] designed UNET-GAN to achieve low-SNR speech enhancement in the time domain. Luo et al. [9] extend the UNET network to the time–frequency domain and perform speech enhancement in the complex audio spectrum. After then, DCCRN [10] introduces the complex feature fusion module into the network to better realize speech time–frequency domain correlation modeling.

The speech signal can receive its corresponding time–frequency spectrum through the short-time Fourier transform (STFT) algorithm [11]. Most speech enhancement algorithms are implemented based on the time–frequency spectrum, which differs from the image enhancement: the time–frequency spectrum is a sequence with causality on the time axis, but images are not time series. Therefore, most speech enhancement models will introduce a recurrent neural network (RNN) as a sequence model to extract the spectral features’ temporal correlation [12]. As a Markov-style sequence model [13], RNN focuses more on modeling the correlation between adjacent elements of the sequence and has strong position awareness, which can better model the correlation between elements and their positions. Those RNN-based models perform well when enhancing speech audios with high SNR. However, when processing low-SNR speech audios, their performance is limited because RNN-based models are challenging to learn long-distance dependencies between sequence elements. At the same time, the model has causal lines when processing sequences and sequence elements that need an input into the model bit by bit, which results in poor parallelizability and high calculation costs [14].

A transformer [14] is a sequence model based on the attention mechanism and can model long-distance dependencies between sequence elements while having good parallelism. The transformer has achieved SOTA in the NLP [15] and CV [16] fields. In this work, we use a transformer module to solve the problem faced by RNNs. However, in the speech enhancement task, the traditional transformer is difficult to deal with complex features. The full attention mechanism is difficult to track the adjacent elements of the sequence, and its computational complexity is also high. The sequence model based on the the attention mechanism has a weak ability to perceive elements and their corresponding positions, and the speech enhancement task requires the model to have a strong enough ability to perceive the positions of the elements. Limited by the above problems, traditional transformer models could be improved in low SNR speech enhancement tasks.

In this paper, we propose a complex sparse transformer (CST) model to improve the transformer in low-SNR speech enhancement tasks. Our contributions are as follows:

1. We propose a transformer-based speech enhancement model suitable for low-SNR scenarios.
2. We extend the transformer operator to the complex domain so that it can efficiently model the correlation between the elements of the real and imaginary parts of the complex sequence features.
3. We improved the transformer module using a sparse attention mask, which has better performance in speech enhancement tasks, while having a lower computational cost.
4. We design the pre-layer positional embedding to enhance the positioning awareness of the transformer model in speech enhancement tasks.

## 2. Related Work

The methods based on neural networks are the current mainstream solution in speech enhancement. The neural network speech enhancement algorithm can be divided into generative and discriminative schemes [17]. The generative model realizes speech enhancement by directly predicting clean speech or using a generation paradigm. Typical methods, such as wave-UNET [7], are the earliest use of the UNET network to generate clean speech from noisy input. SEGAN [18] is a generative adversarial network (GAN) that aims to generate clean speech from noisy speech, and its generator part uses the time–domain UNET structure; the discriminator’s task is judging whether the voice audio generated by the generator is the clean speech corresponding to the noisy speech. Hao et al. [8] design the UNETGAN for low-SNR scenarios; they use dilated convolution to fuse signal temporal features. When using the GAN method to generate clean speech directly, the discriminant indicators are usually tricky to consider the actual speech quality evaluation indicators, and MetricGAN [19] is proposed for this problem so that the model can take into account one or more indicators in the speech quality evaluation metrics to improve the model’s performance from the evaluation metrics’ perspective. Unlike the above models, Soni et al. [20] propose MMSE-GAN; they train a GAN to generate time–frequency spectral masks. Donahue et al. [21] designed a speech enhancement algorithm on the log–Mel spectrum to focus on improving the quality of downstream speech recognition tasks and achieved a 7% word error rate (WER) performance gain by directly generating the log–Mel spectrum corresponding to clean speech.

The effect of the additive background noise on the speech signal can be regarded as the masking of the speech signal by the noise in the time–frequency spectrum. Thus, the speech enhancement model can predict the mask of the noise signal in the time–frequency spectrum or the amplitude spectrum to achieve speech enhancement; such models are called discriminative models. Typical masks include magnitude spectral masks, ideal binary mask (IBM) [22], ideal ratio mask (IRM) [23], and time–frequency spectral masks, complex ratio mask (CRM) [24]. IBM regards the denoising problem as a binary classification problem, using 1 or 0 to mark whether the energy of clean speech dominates the spectral unit. Heymann et al. [25] propose a deep neural network method to predict the IBM. Predicting the IBM is relatively simple; however, it is difficult to accurately describe the proportional energy relationship between clean speech and noise signals in a specific time–frequency unit in complex acoustic scenarios. In order to better distinguish noise in speech audios, Tu et al. [26] propose IRM, which uses a scale to describe the energy ratio relationship between clean speech and noise signals in a spectral unit. Compared with the IBM-based model, the IRM-based model can better model the spectral features of clean speech and eliminate noise more naturally. Strake et al. [27] proposed a two-stage speech enhancement scheme combined with IBM and IRM for low SNR scenarios. In the first stage, a UNET model is used to predict IBM to reduce noise roughly, and a partial convolution model is used in the second stage to repair the loss of speech signal. Both IBM and IRM are masks for the amplitude spectrum of the audio signal. The models that deal with the amplitude spectrum usually assume that the audio signal’s amplitude contains more information than the phase, so the noise reduction is achieved by limiting or eliminating the amplitude of some time–frequency units. To effectively utilize the information in the time–frequency spectrum, Williamson et al. [24] proposes CRM, which describes the proportions of the real part and the imaginary step of the clean speech in the noise-containing frequency signal in the time spectrum unit. Compared to IBM and IRM, the speech enhancement performance through predicting the CRM is better when using the same model structure. To better predict the CRM, Luo et al. [9] proposed DCUNET, which uses the Complex UNET structure to model complex features. Based on DCUNET, DCCRN [10] designed a complex feature fusion module based on long short-term memory (LSTM), which improved the performance of [9]. Among the discriminative models, predictive CRM has become the mainstream method in this field.

RNN-based models such as LSTM and the Gated Recurrent Unit (GRU) are widely used in UNET-based speech enhancement models [28]. However, it is difficult for RNNs to model the long-distance dependence between sequence elements, which limits RNNs’ performance in several tasks, including speech enhancement. In 2017, Vaswani et al. [14] proposed an attention-based transformer model, which can efficiently model long-distance dependencies and, therefore, achieved good results in many fields. Based on the transformer model, the Complex Transformer [29] is defined and applied to the music generation task, which achieves good performance. However, achieving the expected performance improvement in practice is difficult when the transformer structure is directly applied to the speech enhancement task. Kim et al. [30] combine the transformer with a Gaussian model to achieve better performance. Yu et al. [31] add Loca LSTM modules before the multi-head attention to achieve the performance improvement of the transform. Currently, there is no speech enhancement algorithm based on a transformer designed for low-SNR scenarios.

## 3. Method

In this work, we assume that the most important factor affecting speech quality is the additive background noise, so the noisy speech can be expressed as:

(1) y(i)=s(i)+n(i)

Among them, y(i), s(i), and n(i) represent the *i* frame of the noisy speech, clean speech, and noise, respectively. We aim to estimate $s^{\prime }$ from *y* as close as possible to the origin *s*.

**Figure 1:**
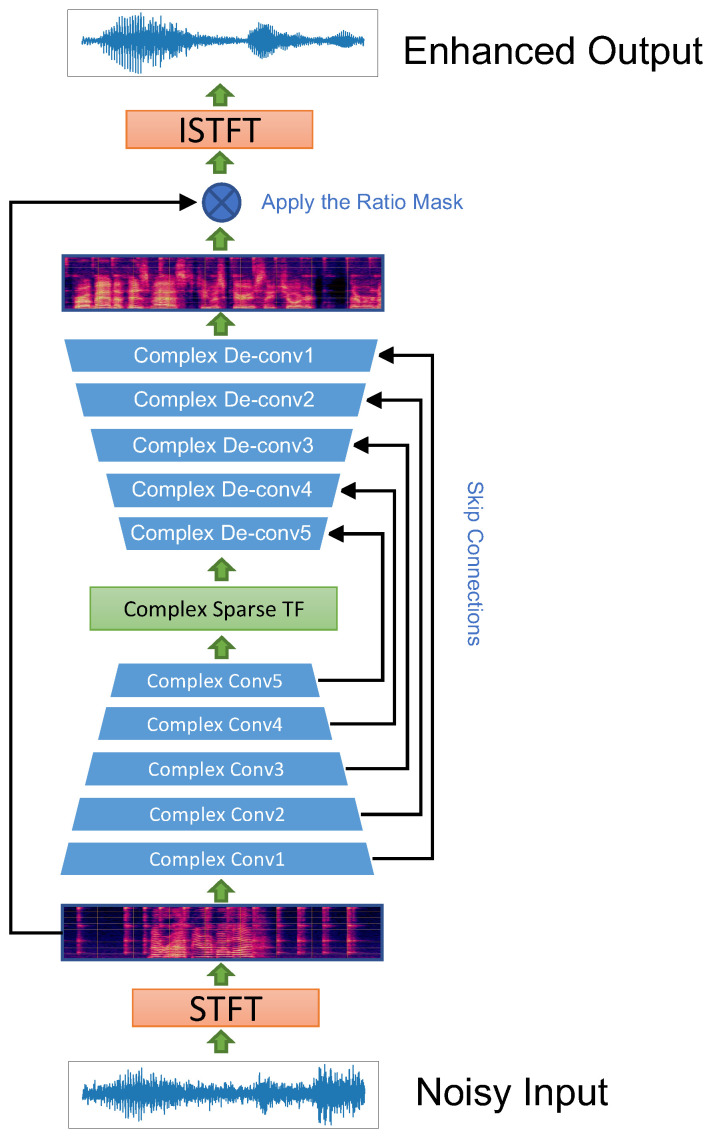
Model overall structure.

In this work, we propose the CST network, which uses the complex convolution and deconvolution modules in the form of UNET as the codec to extract and restore the high-level features of the spectrum of speech; it uses the CST module to fuse and edit the complex audio features; the background noise removal is achieved by predicting the CRM. The basic structure of this model is shown in Figure 1. The first part of this chapter will introduce the basic architecture of this model, and the second part will introduce our CST module.

### 3.1. Model Basic Structure

**Figure 2:**
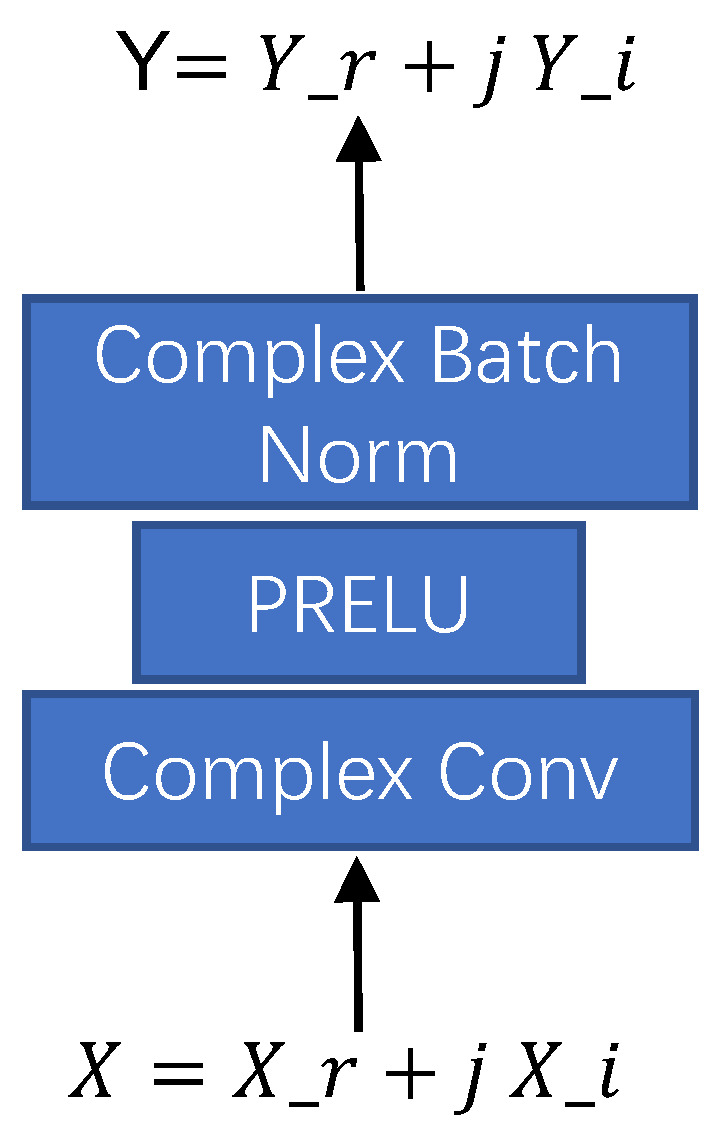
Complex convolution module.

Our model acts on the complex time–frequency spectrum and uses complex convolution and deconvolution modules to extract and restore the time–frequency spectrum features of speech audios. The basic structure of the complex convolution module is shown in Figure 2; the deconvolution module is similar to the convolution module. Those modules accept complex feature inputs and output complex features. The complex convolution operation can be expressed as:

(2) $$\begin{array}{cc} ComplexConv(X)= & \left(Y_{rr}-Y_{ii}\right)+j\left(Y_{ir}+Y_{ri}\right)\\ Y_{rr}= & Conv_{r}\left(X_{r}\right),Y_{ii}=Conv_{i}\left(X_{i}\right)\\ Y_{ir}= & Conv_{i}\left(X_{r}\right),Y_{ri}=Conv_{r}\left(X_{i}\right) \end{array}$$

In the formula, *X* and *Y* are complex features representing the model’s input and output, respectively. $Conv_{r}\left(*\right),Conv_{i}\left(*\right)$ are real convolution and imaginary convolution, respectively. The complex convolution and deconvolution modules consist of a complex convolution (deconvolution) layer, a PRelu layer, and a Complex Batch Normalization layer. The codec based on the complex residual module is distributed in an inverted pyramid, and the skip-layer links from the basic structure of the UNET architecture network.

The model achieves speech enhancement by predicting the CRM, which removes noise by predicting and learning to scale the noise component on the complex time spectrum. This process can be expressed as follows:

(3) $$\hat{X}=X_{r}\cdot CRM_{r}+j\left(X_{i}\cdot CRM_{i}\right)$$

In the formula, CRM is the complex ratio mask, *X* is the model’s input, and $\hat{X}$ is the model’s prediction of the spectrum of the clean speech signal. After the inverse short-time Fourier transform, the corresponding time domain signal can be obtained. This model uses the Scaled Invariant SNR (SI-SNR) as the loss function, which can be expressed as:

(4) $$\begin{array}{cc} S_{target} & =\frac{<\hat{s},s>}{{\left||s|\right|}_{2}^{2}}s\\ S_{noise} & =\hat{s}-s_{target}\\ SI-SNR & =10log10(\frac{\left|\right|s_{target}{\left|\right|}_{2}^{2}}{\left|\right|s_{noise}{\left|\right|}_{2}^{2}}) \end{array}$$

where <∗,∗> represents the vector dot product, ${\left||*|\right|}_{2}^{2}$ represents the l2 norm, $\hat{s}$ represents the audio time domain signal predicted by the model, and *s* represents the clean speech signal.

### 3.2. Complex Sparse Transformer

The CST module is designed to fuse and edit the complex feature of speech audios extracted by the complex UNET codec. To allow the transformer structure to process complex features, we regard the transformer as an overall operator and use complex multiplication rules to fuse the real and imaginary features of the complex field. The process can be expressed as follows:

(5) $$\begin{array}{cc} X & =X_{r}+jX_{i}\\ Y & =(Transformer_{r}\left(X_{r}\right)-Transformer_{i}\left(X_{i}\right))\\ & +j(Transformer_{r}\left(X_{i}\right)-Transformer_{i}\left(X_{r}\right)) \end{array}$$

**Figure 3:**
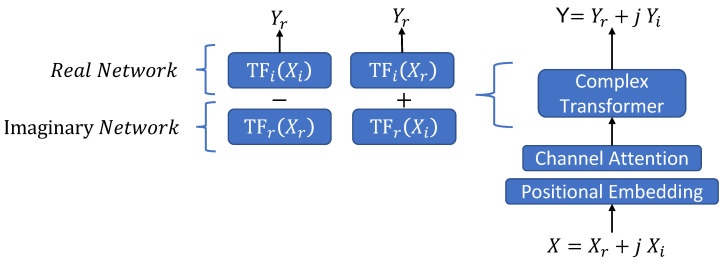
Complex transformer architecture.

Figure 3 shows the complex domain transformer structure. Each complex transformer module layer contains two transferormer sub-modules, $Transformer_{r}\left(*\right)$ and $Transformer_{i}\left(*\right)$, representing the real number network and the imaginary number network, respectively. From past research, there is another way to expend the transformer for complex features, which uses the ternary complex number operation rules to calculate the attention matrix; our early experiments show our way is more effective.

**Figure 4:**
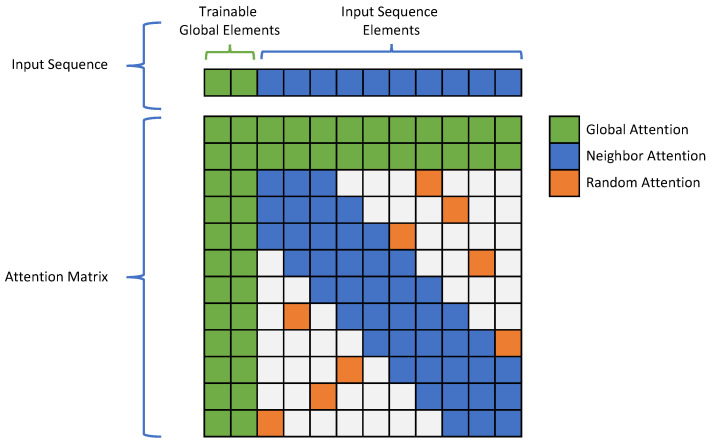
Sparse attention mask.

Short-range correlations between speech signals are stronger than long-range, so it is necessary to introduce this prior knowledge into the model. The RNN-based models receive the elements in the sequence one by one; in this process, the information farther away from the current node will be more forgotten, and such a structure conforms to the characteristic of the speech signal. However, the complete attention transformer will not be affected by the distance between elements when generating the attention matrix, which can model the correlation between elements at any distance. However, at the same time, it will not be able to allocate enough attention between elements at short-distance correlations. To enhance the transformer module, we introduce a sparse attention mask [32] to balance the model’s attention to the relationship between long-range and short-range dependencies. The attention computation process with a mask can be expressed as:

(6) $$\begin{array}{cc} mask & \in {\left\{0,1\right\}}^{n\times n}\\ SpaarseAttention(Q,K,V) & =\left(softmax\left(\frac{QW^{Q}{\left(KW^{K}\right)}^{⊺}}{\sqrt{d_{k}}}\right)\cdot mask\right)VW^{V}W^{O} \end{array}$$

where *n* is the length of the sequence *V*, and the mask matrix mask is a binary matrix with values of 0 and 1. When $mask_{i,j}=1$, the *i*th element of *V* sequence will generate attention to the *j*th element; otherwise, if $mask_{i,j}=0$ the *i*th element of the *V* sequence will not notice the *j*th element. Figure 4 shows the attention mask we use.

Our attention mask mainly consists of global, neighbor, and random attention. Global attention corresponds to a group of particular elements at the head of the sequence, they can be associated with elements in the whole sequence within the attention calculation, and elements in the whole sequence can also be associated with them. In order to avoid head elements in the sequence feature process undertaking different training tasks from other elements, the sequence elements corresponding to the global attention are trainable parameters in this model that will be concatenated into the head of the input features before the calculation of the attention mechanism, and will be discarded after the calculation. Neighbor attention means that each element can associate with the fixed multi-elements around it. Random attention is randomly distributed in the attention mask to accelerate the establishment of association relationships. Our sparse attention mask reduces the space complexity of the attention matrix from $O(N^{2})$ to O(N), which greatly reduces the computational cost of the model during training and inference, and the experimental results are discussed in Section 4.3.

**Figure 5:**
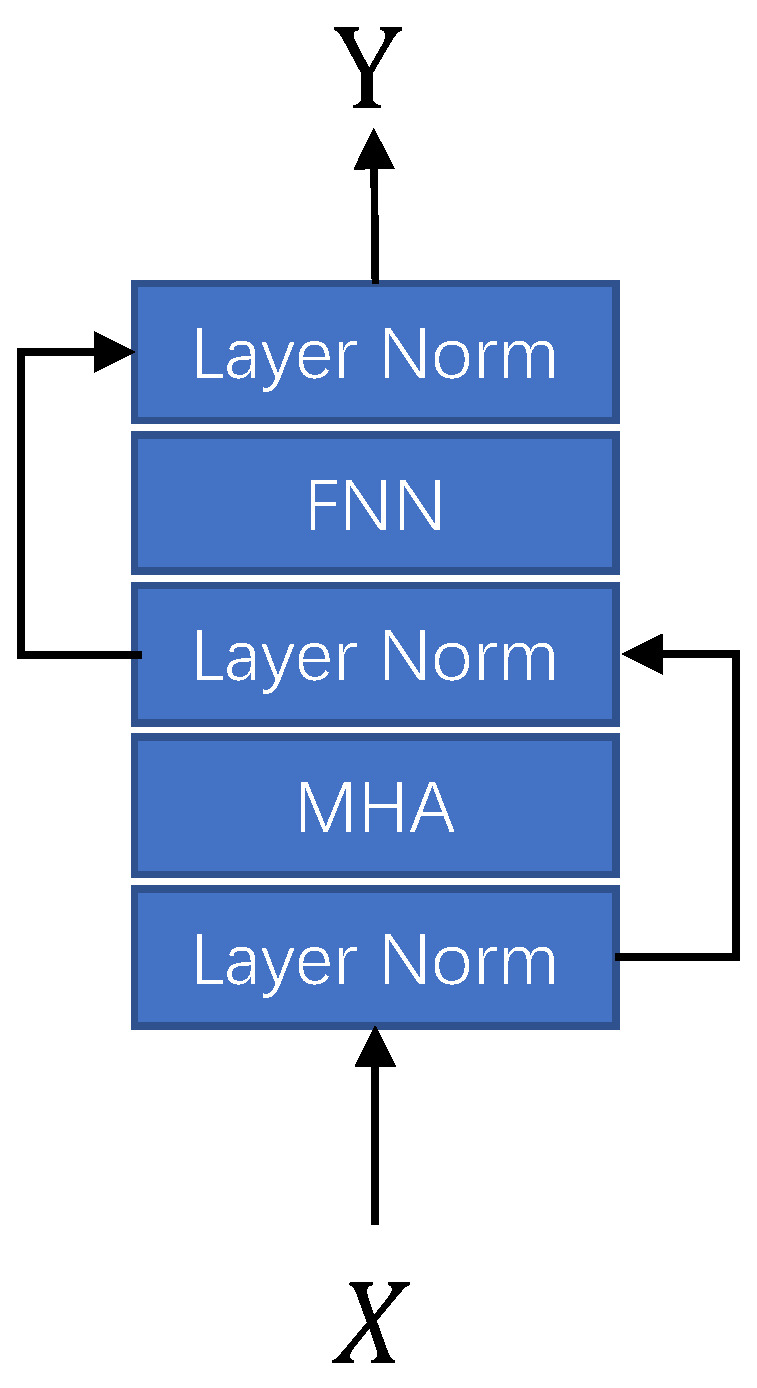
Inner structure of complex sparse transformer.

The transformer model is poorer than RNN and Temporal Convolutional Network (TCN) in time sequence modeling tasks. Zeng et al. [33] believe that the main reason is that the transformer has a weak ability to perceive the position and sequence of elements in the sequence, so there is a modeling bottleneck. In this paper, our technical route to achieve speech enhancement is to remove the noise contained in the audio by predicting the mask, and this task has high requirements on the model’s positioning awareness and modeling ability. Therefore, we introduce the form of pre-layer positional embedding to enhance the location awareness of the transformer model. To cooperate with the new structure of the layer-by-layer injection of positional embedding, we adjusted the transformer’s internal structure. We put the layer normalization module in front to avoid the deviation of feature statistics introduced by the additive positional. The module structure is shown in Figure 5. Regarding selecting positional embedding schemes, we implement a scheme based on sine functions and a scheme based on trainable parameters, the differences between the two are discussed in Section 4.3.

**Figure 6:**
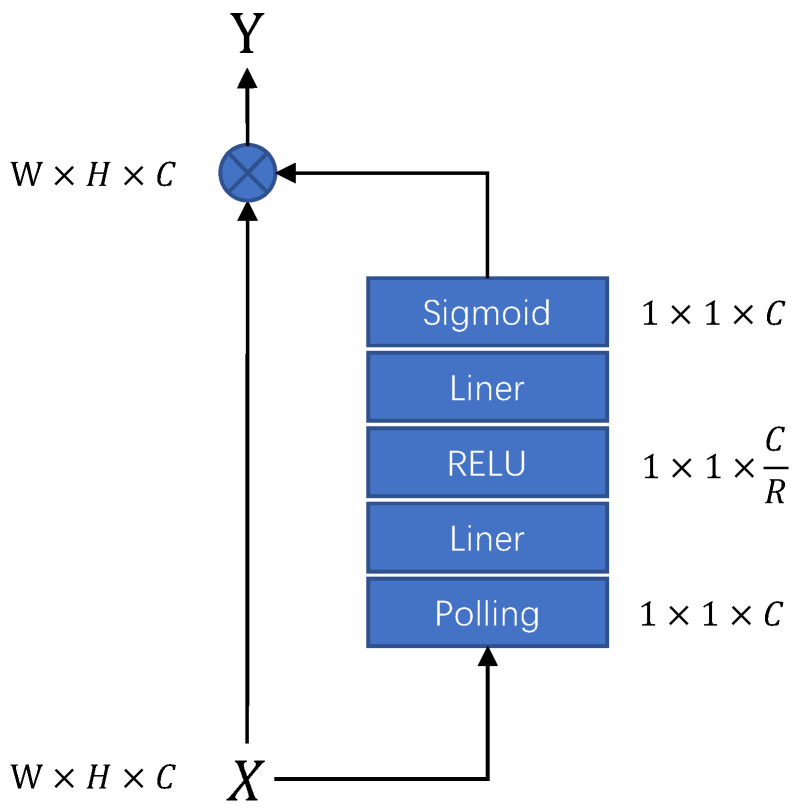
Channel attention architecture.

The convolution module extracts the audio features from the frequency dimension, and the energy of the speech signal is mainly concentrated in the low-frequency band. However, in the speech enhancement task, especially the low-SNR speech enhancement task, the low-frequency audio signals may be masked and confused by the noise. Therefore, we hope the model can fuse convolutional features from different channels of convolutional features and assign weights to achieve more effective feature preprocessing for different kinds of noisy audio. To achieve this goal, we use the Squeeze-and-Excitation [34] module as a channel attention for feature enhancement in the channel dimension. The basic structure of the channel attention module is shown in Figure 6.

## 4. Experiment

In this paper, we use the MUSAN [35] clean speech and noise dataset, CSTR’s VCTK Corpus [36] (Centre for Speech Technology Voice Cloning Toolkit) clean speech dataset, NoiseX92 [37] noise dataset, and Babble noise data [38] for experiments. The MUSAN dataset contains 60 h of speech and 48 h of music and noise data. We use all clean speech data and treat all 48 h of noise and music data as noise data. We divide the speech and noise data in the MUSAN dataset into training, verification, and test sets according to the ratio of 8:1:1 and ensure that the three do not intersect. In order to improve data diversity and avoid overfitting, we randomly sample a piece of voice data from the training set during each training and verification process, a piece of random noise with the same length as the noisy speech, and use a random SNR between [−15 dB and 15 dB] to dynamically synthesize the noisy speech; for the test set, we randomly select a piece of noise data for each clean speech in the test set and select a random SNR between [−15, −10, −5, and 0] dB to synthesize each noisy speech. VCTK is a large English speech dataset that includes more than 400 sentences by 100 speakers’ and is widely used in speech synthesis and speech enhancement tasks. The NoiseX92 dataset contains a set of stationary noises collected in real scenes, such as factory floors, engine rooms, inside vehicles, etc. The Babble contains an hour of noise data from MyNoise that simulates the noisy environment of a cafe and has more complex components. It mainly includes messy and irregular human voices and cafe-specific cutlery, knife and fork collision, etc. The noise components are more complex and similar to speech signals in energy distribution which causes it to be more likely to be confused with speech signals.

**Figure 7:**
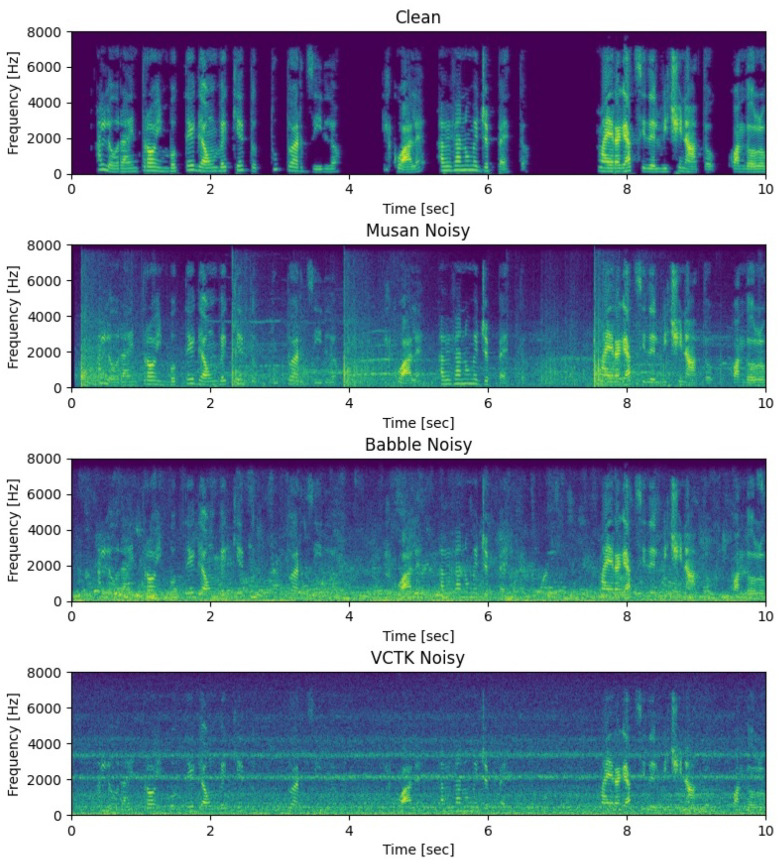
Noisy speech in three datasets.

We set up three different test sets to evaluate the performance of the model. The MUSAN test set includes a variety of environmental noises and various types of music. We use this dataset to evaluate the noise reduction ability of the model for different types of single-type noise. The Babble test set is based on the noise data in the Babble dataset and the clean speech in MUSAN. Since the Babble noise is similar in energy distribution to speech signals, we use this dataset to evaluate the model’s anti-interference ability. The VCTK test set is synthesized from VCTK clean speech and NoiseX92 noise data. This test set is used to evaluate the ability of the model to remove stationary noise. All three test sets are synthesized with the same SNR distribution. Typical audio magnitude spectra in the two test sets are shown in Figure 7.

We use Perceptual Evaluation of Speech Quality (PESQ) and Short-Time Objective Intelligibility (STOI) as objective metrics to evaluate the performance of all models. PESQ is recommended by the ITU Telecommunication Standardization Sector (ITU-T), which is a widely-used full-reference speech quality metric with values ranging from −0.5 to 4.5. A higher PESQ value indicates better speech quality. STOI, on the other hand, is used to measure speech intelligibility, with values ranging from 0 to 1. A higher STOI value indicates better intelligibility. To ensure compatibility with these metrics, we divide long audio samples into 10-second segments and discard segments containing less than 50% speech signals.

In the codec of our models, we use five layers of complex convolution and deconvolution modules separately. Each processing audio length is 8192 frames. In the short-time Fourier transform, we use a Hanning window with a length of 512 and a moving width of 128. In training, each batch contains 16 voice clips, the optimizer is adamW [39], the learning rate is 0.0004, the beta1 is 0.9, and the beta2 is 0.999. The training platform uses a single RTX3090.

We designed three experiments in this work. In the first experiment, we compared our model with other well-established speech enhancement models. In the second experiment, we explored the effect of the number of layers on the model’s performance. In the last experiment, we investigated the effectiveness of the improvements we implemented to the model through ablation experiments.

### 4.1. Compare with Other Models

In this section, we compared our models with four typical speech enhancement models, DCCRN [10], CRN [40], SEGAN [18], and SEFORMER [31]. Regarding model structure, the basic structure of DCCRN, CRN, and SEGAN is the UNET structure, and SEFORMER is the transformer decoder structure. Specifically, DCCRN uses a complex UNET network to enhance speech by predicting the CRM, which is one of the SOTAs in the DNS challenge board list. CRN is a real number model that realizes speech enhancement by predicting the IRM, and this model is a widely recognized classic model in the field of speech enhancement. SEGAN is a GAN-based generative speech enhancement model that achieves speech enhancement by generating target audio from noisy audio. SEFORMER is a pure transformer structure speech enhancement model improved for speech enhancement tasks. This model uses the Local-LSTM structure to solve the problem of the lack of location awareness of the transformer model, thus endowing the transformer model with the ability to handle speech tasks. SEFORMER is a discriminative model for speech enhancement by predicting IRM. All the above models are implemented as described in the original text. In order to enhance the performance of the SERFORMER model, four layers of SEFORMER were used in our experiments for comparison.

The comparative experimental results in the Musan, Babble, and VCTK test sets are shown in Table 1, Table 2 and Table 3, respectively. In the table, Noisy represents noisy speech, and DCCRN, CRN, SEGAN and SEFORMER represent three models; CST-8 and CST-16 are the models proposed in this paper, representing eight layers and sixteen layers, respectively.

The experimental results in three test sets show that our proposed model, CST-8 and CST-16, outperforms the other three models in terms of both PESQ and STOI average metrics. In the MUSAN test set, our model improved the PESQ by 29.6% and the STOI by 9.7% compared to the noisy speech and outperformed DCCRN, the best-performing model among the control group, by 4.1% and 1.8%, respectively. In the Babble test set, our model improved by 11.9% on PESQ and 4.7% on STOI compared to the noisy speech. Our model also outperformed DCCRN by 2.8% on PESQ and 1.1% on STOI. In the VCTK test set, our model has dramatically improved the speech quality compared with the original noise. Regarding speech clarity, our model has improved by 21.88% compared with the original speech on PESQ. In terms of the intelligibility, compared with noisy speech, it improves by 2.29% on STOI. Among the comparison models, DCCRN has the best overall performance. Compared with this model, our model has improved by 7.53% on PESQ, but it is comparable to STOI.

In the Babble test set −15 dB task, our model has a decline in clarity and intelligibility compared to the original noisy speech; SEFORMER has the best inference results in this task and surpasses our speech audio and noisy in the intelligibility metric. In the VCTK test set −15 dB task, although our speech has improved the most in terms of clarity compared with other models, it is not as good as SEFORMER in terms of intelligibility and has declined compared to the original Noisy. On the VCTK −10 dB task, our model performs slightly worse than SEFORMER in terms of speech intelligibility.

Overall, this model performs better than other models in SNR scenarios and is more suitable for scenarios with various types of noise. However, in the case of an extremely low signal-to-noise ratio, the adaptability of this model to the noise type is speech babble, or else a low-frequency stationary noise scene is relatively insufficient.

### 4.2. Effect of Layers

The number of layers in a deep learning model can significantly impact its performance. As the number of layers increases, the model’s ability to represent complex relationships increases, allowing it to fit more intricate mapping functions. However, the relationship between the number of layers and performance is not always linear. When the number of layers reaches a certain point, adding more layers may no longer provide performance gains and can even decrease performance. In this experiment, we explored the effect of the number of transformer model layers on the performance and provided recommendations for using this model.

We use a sparse transformer model to model audio features to establish dependencies between elements in the sequence; at least two layers are required to associate with every two elements. Therefore, we use a 4-layer transformer as a starting point and experiment with 4-, 8-, 12-, and 16-layer transformer models on MUSAN, Babble, and VCTK test sets. The results are shown in Table 4, Table 5 and Table 6, respectively.

As shown in Table 4, in the MUSAN test set, the four-layer model already has good basic performance. Compared with the original noisy speech, the speech quality has been greatly improved, the PESQ has been improved by 25.02% and 8.31% on STOI. Compared with the four-layer model, the eight-layer model also has an obvious improvement in speech quality, among which the improvement in PESQ is 3.59%, and the improvement in STOI is 1.20%. However, compared with the 8th layer, the 12th layer only has a small improvement in speech clarity, which is shown as an increase of 0.80% in the PESQ, and the 16th layer and the 12th layer have the same speech quality. Regarding speech clarity, the 12-layer model has the best performance, which is 30.54% higher than the noisy speech, and 4.41% higher than the 4-layer model. Regarding intelligibility, the 16-layer model has the best performance, which is 9.74% higher than the noisy speech, and 1.32% higher than the 4-layer model.

As shown in Table 5, the four-layer model also has a strong basic performance in the Babble test set. It has improved by 8.99% in PESQ compared with noisy speech and 3.02% in STOI. Compared with the four-layer model, the speech quality of the eight-layer model has slightly improved: it has improved PESQ by 2.67% and STOI by 1.69%. However, the 12-layer and 16-layer models failed to improve the performance over the 8-layer model in this task. In this test set, the 8-layer model has the best performance. Compared with noisy speech, PESQ has increased by 11.91% and STOI has increased by 4.76%.

As shown in Table 6, in the VCTk test set, the 4-, 8-, 12-, and 16-layer models have no significant difference in performance. In terms of speech intelligibility, the best model is the 12-layer model, which is relatively noisy speech. It has improved by 22.01% on PESQ, and the best intelligibility is the four-layer model, which has improved by 2.29% on STOI compared to noisy speech.

Overall, in the scenes with diverse noise types, more layers mean better performance, but increasing the number of layers beyond 12 layers no longer yields significant performance gain. For the scene where speech babble is the prominent background noise, the eight-layer model is almost at the performance ceiling. For stationary noise scenes, the four-layer model can produce better results, and the performance of increasing the number of layers does not improve significantly.

### 4.3. Ablation Experiments

We designed ablation experiments to explore the impact of sparse attention masks, pre-layer positional embedding, and channel attention on model performance. The previous experiment has proved that when the number of layers of the model is more than eight, increasing the number of layers can no longer significantly improve the model’s performance. Therefore, we only conduct research based on the eight-layer transformer in the ablation experiment. The results of the ablation experiments are shown in Table 7, Table 8 and Table 9.

In the above tables, NO-TF indicates a complex UNET model without any feature fusion module in the middle, TF indicates the full-attention transformer model, +MASK indicates the transformer model with a sparse attention mask, +SIN and +TRAIN indicate the addition of a sine function for pre-layer positional embedding and trainable parameters as positional embedding, and +CA indicates the introduction of channel attention.

As shown in Table 7, in the MUSAN test set, compared with NO-TF, after adding a Complex Transformer as a feature fusion module, the speech quality has a significant improvement in clarity and intelligibility, among which PESQ has increased by 4.22%; the STOI increased by 2.68%. Compared with TF, after adding the sparse attention mask, the speech intelligibility decreases slightly, and the intelligibility improves slightly; the introduction of the pre-layer positional embedding and channel attention improves both PESQ and STOI indicators. In this test set, the best combination in terms of speech intelligibility is TF + MASK + SIN + CA, and its PESQ has increased by 4.47% compared with NO-TF; compared with TF, it has increased by 1.24%; in terms of speech intelligibility, the best combination is TF + MASK + TRAIN + CA, its STOI increased by 3.83% compared with NO-TF, and increased by 1.12% compared with TF.

As shown in Table 8, in the Babble test set, compared with NO-TF, the speech quality has been significantly improved after the introduction of the feature fusion module, of which the PESQ has increased by 4.22%; the STOI has increased by 2.68%. After adding the sparse attention mask, the speech quality is almost unchanged. The introduction of pre-layer positional embedding and channel attention has slightly improved the speech quality. In this test set, the best combination is TF + MASK + TRAIN + CA; compared with NO-TF, it has improved by 4.47% on PESQ, and on STOI increased by 3.83%. Compared with TF, it has increased by 1.24% on PESQ and 1.12% on STOI.

As shown in Table 9, in the VCTK test set, compared with NO-TF, the introduction of the feature fusion module has improved speech clarity, and the speech intelligibility is comparable. After adding the attention mask, both the clarity and intelligibility are improved. Compared with NO-TF, the PESQ is improved by 2.94%, and the STOI is improved by 0.42%. The introduction of layer-wise positional encoding and channel attention in this test set has no significant performance improvement. In this task, the best combination is TF-MASK, which improves the PESQ by 1.41% and the STOI by 0.67% compared to TF.

In general, after introducing CST as a feature fusion module, the model’s performance has been improved in the three test sets, and the performance improvement in the MUSAN and Babble test sets corresponding to non-stationary noise is relatively more significant. In different CST implementations, compared with the full attention model, the model using sparse attention has improved speech intelligibility in non-stationary noise scenes and improved speech quality in stationary noise scenes. We believe this proves our hypothesis in Section 3.2: short-range correlations of speech signals are stronger than long-range correlations. On the other hand, as shown in Table 10, the introduction of the sparse attention matrix significantly reduces the computational cost of the model. The complexity of the attention matrix is reduced from $O(N^{2})$ to O(N), and IT reduced THE GPU memory usage during training by 50%. The introduction of pre-layer positional embedding and channel attention strengthens the model’s ability to model non-stationary noise, but there is no significant improvement in stationary noise scenarios; in terms of pre-layer positional embedding scheme selection, the trainable parameter scheme has a relatively better Effect.

## 5. Conclusions

We designed the CST model for speech enhancement tasks in low-SNR scenarios in this work. Compared with previous RNN-based network models and transformer-based models, our model can better model long-distance dependencies between audio features. Compared with the original transformer structure, the attention distribution in our model is more suitable for processing speech features and has a lower computational cost; the pre-layer positional embedding and the channel attention modules strengthen the transformer’s speech signal processing capabilities. The experimental results show that our model can better remove the background noise contained in the audio in the low-SNR scene and has a remarkable improvement in the STOI and PESQ metrics.

At present, our model uses a multi-layer transformer structure. Although the sparse attention mechanism reduces the computational cost compared with the original transformer, the computational cost is still too high for devices with limited computing power, such as edge terminals. In future work, we will reduce the calculation amount of the model and compress the model size using distillation, quantization, and pruning to achieve efficient reasoning on devices with limited computing power.

## Figures and Tables

**Table 1:** Compare performance with other models in the MUSAN test set.

Model Name	PESQ				STOI			
	−15 dB	−10 dB	−5 dB	0 dB	−15 dB	−10 dB	−5 dB	0 dB
Noisy	1.512	1.634	1.819	2.101	0.631	0.717	0.792	0.857
DCCRN [10]	1.736	2.035	2.330	2.691	0.695	0.783	0.850	0.902
CRN [40]	1.510	1.644	1.837	2.123	0.643	0.731	0.806	0.863
SEGAN [18]	1.392	1.460	1.623	1.789	0.532	0.625	0.728	0.794
SEFORMER [31]	1.541	1.661	1.855	2.143	0.631	0.717	0.792	0.857
CST-8	1.808	2.117	2.439	**2.787**	0.712	0.799	0.863	0.911
CST-16	**1.815**	**2.124**	**2.440**	2.778	**0.715**	**0.800**	**0.863**	**0.911**

**Table 2:** Compare performance with other models in the Babble test set.

Model Name	PESQ				STOI			
	−15 dB	−10 dB	−5 dB	0 dB	−15 dB	−10 dB	−5 dB	0 dB
Noisy	1.383	1.396	1.532	1.761	0.467	0.589	0.725	0.804
DCCRN [10]	1.278	1.470	1.759	2.097	0.448	0.614	0.770	0.843
CRN [40]	1.331	1.473	1.537	1.778	0.465	0.595	0.742	0.825
SEGAN [18]	1.293	1.345	1.544	1.764	0.448	0.580	0.721	0.784
SEFORMER [31]	**1.342**	1.436	1.560	1.788	**0.497**	0.625	0.753	0.810
CST-8	1.311	**1.509**	**1.822**	**2.153**	0.453	**0.625**	**0.779**	**0.851**
CST-16	1.305	1.505	1.801	2.140	0.453	0.622	0.775	0.849

**Table 3:** Compare performance with other models in the VCTK test set.

Model Name	PESQ				STOI			
	−15 dB	−10 dB	−5 dB	0 dB	−15 dB	−10 dB	−5 dB	0 dB
Noisy	1.255	1.299	1.407	1.615	0.494	0.549	0.618	0.692
DCCRN [10]	1.283	1.410	1.657	1.970	0.486	**0.562**	0.644	0.720
CRN [40]	1.231	1.200	1.194	1.208	0.432	0.451	0.475	0.503
SEGAN [18]	1.294	1.329	1.421	1.618	0.450	0.508	0.588	0.672
SEFORMER [31]	1.247	1.303	1.435	1.659	**0.493**	0.549	0.618	0.691
CST-8	1.326	1.504	1.799	2.130	0.477	0.556	0.644	0.722
CST-16	**1.337**	**1.515**	**1.801**	**2.143**	0.480	0.559	**0.645**	**0.723**

**Table 4:** Effect of different layers in the MUSAN test set.

Model Name	PESQ				STOI			
	−15 dB	−10 dB	−5 dB	0 dB	−15 dB	−10 dB	−5 dB	0 dB
Noisy	1.512	1.634	1.819	2.101	0.631	0.717	0.792	0.857
CST-4	1.761	2.050	2.344	2.679	0.702	0.788	0.852	0.904
CST-8	1.808	2.117	2.439	2.787	0.712	0.799	0.863	0.911
CST-12	1.814	**2.133**	**2.460**	**2.817**	0.711	0.796	0.861	0.909
CST-16	**1.815**	2.124	2.440	2.778	**0.715**	**0.800**	**0.863**	**0.911**

**Table 5:** Effect of different layers in the Babble test set.

Model Name	PESQ				STOI			
	−15 dB	−10 dB	−5 dB	0 dB	−15 dB	−10 dB	−5 dB	0 dB
Noisy	1.383	1.396	1.532	1.761	0.467	0.589	0.725	0.804
CST-4	1.310	1.490	1.753	2.065	0.441	0.612	0.768	0.842
CST-8	**1.311**	**1.509**	**1.822**	**2.153**	**0.453**	**0.625**	**0.779**	**0.851**
CST-12	1.310	1.486	1.787	2.128	0.442	0.618	0.772	0.848
CST-16	1.305	1.505	1.801	2.140	0.453	0.622	0.775	0.849

**Table 6:** Effect of different layers in the VCTK test set.

Model Name	PESQ				STOI			
	−15 dB	−10 dB	−5 dB	0 dB	−15 dB	−10 dB	−5 dB	0 dB
Noisy	1.255	1.299	1.407	1.615	0.494	0.549	0.618	0.692
CST-4	1.348	1.513	1.778	2.096	**0.482**	**0.560**	0.645	0.721
CST-8	1.326	1.504	1.799	2.130	0.477	0.556	0.644	0.722
CST-12	**1.350**	**1.521**	**1.805**	2.127	0.479	0.559	0.645	0.720
CST-16	1.337	1.515	1.801	**2.143**	0.480	0.559	**0.645**	**0.723**

**Table 7:** Ablation experiments in the MUSAN test set.

Model Name	PESQ				STOI			
	−15 dB	−10 dB	−5 dB	0 dB	−15 dB	−10 dB	−5 dB	0 dB
Noisy	1.512	1.634	1.819	2.101	0.631	0.717	0.792	0.857
NO-TF	1.741	2.016	2.293	2.643	0.696	0.781	0.845	0.890
TF	1.795	2.098	2.416	2.771	0.707	0.794	0.859	0.908
TF + MASK	1.787	2.092	2.411	2.745	0.712	0.797	0.860	0.910
TF + MASK + SIN	1.791	2.098	2.423	2.777	0.710	0.796	0.861	0.911
TF + MASK + SIN + CA	**1.811**	**2.126**	**2.442**	**2.801**	0.711	0.797	0.861	0.910
TF + MASK + TRAIN	1.801	2.102	2.426	2.771	0.711	0.795	0.861	0.909
TF + MASK + TRAIN + CA	1.808	2.117	2.439	2.787	**0.712**	**0.799**	**0.863**	**0.911**

**Table 8:** Ablation experiments in the Babble test set.

Model Name	PESQ				STOI			
	−15 dB	−10 dB	−5 dB	0 dB	−15 dB	−10 dB	−5 dB	0 dB
Noisy	1.383	1.396	1.532	1.761	0.467	0.589	0.725	0.804
NO-TF	1.278	1.467	1.697	1.998	0.440	0.596	0.746	0.826
TF	1.309	1.496	1.788	2.119	0.444	0.618	0.770	0.846
TF + MASK	1.284	1.489	1.785	2.129	0.451	0.623	0.776	0.848
TF + MASK + SIN	1.285	1.496	1.800	2.134	0.448	0.621	0.772	0.848
TF + MASK + SIN + CA	1.300	1.496	1.799	2.133	0.445	0.617	0.770	0.846
TF + MASK + TRAIN	1.310	1.486	1.787	2.128	0.442	0.618	0.772	0.848
TF + MASK + TRAIN + CA	**1.311**	**1.509**	**1.822**	**2.153**	**0.453**	**0.625**	**0.779**	**0.851**

**Table 9:** Ablation experiments in the VCTK test set.

Model Name	PESQ				STOI			
	−15 dB	−10 dB	−5 dB	0 dB	−15 dB	−10 dB	−5 dB	0 dB
Noisy	1.255	1.299	1.407	1.615	0.494	0.549	0.618	0.692
NO-TF	1.298	1.452	1.738	2.070	0.479	0.558	0.645	0.722
TF	1.328	1.486	1.760	2.083	0.480	0.557	0.642	0.719
TF-MASK	**1.336**	**1.507**	1.789	2.119	**0.484**	**0.561**	**0.646**	**0.723**
TF + MASK + SIN	1.300	1.461	1.749	2.088	0.474	0.554	0.643	0.720
TF + MASK + SIN + CA	1.286	1.440	1.730	2.080	0.474	0.554	0.642	0.721
TF + MASK + TRAIN	1.306	1.470	1.754	2.081	0.470	0.551	0.640	0.718
TF + MASK + TRAIN + CA	1.326	1.504	**1.799**	**2.130**	0.477	0.556	0.644	0.722

**Table 10:** Computational cost of different kinds of CST implements.

Model Name	Parameter Count	Attention Complexity	Training GPU	Inferencing GPU
			Memory Usage	Memory Usage
NO-TF	2.6 M	-	3443 MB	1304 MB
TF	38.6 M	$O(N^{2})$	17,482 MB	1650 MB
TF-MASK	38.4 M	O(N)	8796 MB	1526 MB
TF + MASK + SIN	38.4 M	O(N)	8788 MB	1542 MB
TF + MASK + SIN + CA	42.6 M	O(N)	9012 MB	1558 MB
TF + MASK + TRAIN	42.6 M	O(N)	8988 MB	1542 MB
TF + MASK + TRAIN + CA	46.8 M	O(N)	8834 MB	1558 MB

## Data Availability

This work uses three open-source audio datasets (VCTk [36], MUSAN [35], and NoiseX92 [37]) and a private noise dataset from MyNoise [38]. All open-source datasets are available online, and the MyNoise data need to get permission from the website owner.

## References

[1] Das N., Chakraborty S., Chaki J., Padhy N., Dey N. (2021). Fundamentals, present and future perspectives of speech enhancement. Int. J. Speech Technol..

[2] Hao X., Su X., Horaud R., Li X. Fullsubnet: A full-band and sub-band fusion model for real-time single-channel speech enhancement. Proceedings of the ICASSP 2021—2021 IEEE International Conference on Acoustics, Speech and Signal Processing (ICASSP).

[3] Zheng C., Peng X., Zhang Y., Srinivasan S., Lu Y. Interactive speech and noise modeling for speech enhancement. Proceedings of the AAAI Conference on Artificial Intelligence.

[4] Lu X., Tsao Y., Matsuda S., Hori C. (2013). Speech Enhancement Based on Deep Denoising Autoencoder. Interspeech.

[5] Ronneberger O., Fischer P., Brox T. (2015). U-net: Convolutional networks for biomedical image segmentation. Proceedings of the International Conference on Medical Image Computing and Computer-Assisted Intervention.

[6] Stoller D., Ewert S., Dixon S. (2018). Wave-u-net: A multi-scale neural network for end-to-end audio source separation. arXiv.

[7] Macartney C., Weyde T. (2018). Improved speech enhancement with the wave-u-net. arXiv.

[8] Hao X., Su X., Wang Z., Zhang H. (2020). UNetGAN: A robust speech enhancement approach in time domain for extremely low signal-to-noise ratio condition. arXiv.

[9] Luo Y., Mesgarani N. (2019). Conv-tasnet: Surpassing ideal time–frequency magnitude masking for speech separation. IEEE/ACM Trans. Audio Speech Lang. Process..

[10] Hu Y., Liu Y., Lv S., Xing M., Zhang S., Fu Y., Wu J., Zhang B., Xie L. (2020). DCCRN: Deep complex convolution recurrent network for phase-aware speech enhancement. arXiv.

[11] Griffin D., Lim J. (1984). Signal estimation from modified short-time Fourier transform. IEEE Trans. Acoust. Speech Signal Process..

[12] Weninger F., Erdogan H., Watanabe S., Vincent E., Roux J.L., Hershey J.R., Schuller B. (2015). Speech enhancement with LSTM recurrent neural networks and its application to noise-robust ASR. Proceedings of the International Conference on Latent Variable Analysis and Signal Separation.

[13] Zhao J., Huang F., Lv J., Duan Y., Qin Z., Li G., Tian G. Do rnn and lstm have long memory?. Proceedings of the International Conference on Machine Learning. PMLR.

[14] Vaswani A., Shazeer N., Parmar N., Uszkoreit J., Jones L., Gomez A.N., Kaiser Ł., Polosukhin I. Attention is all you need. Proceedings of the Advances in Neural Information Processing Systems 30 (NIPS 2017).

[15] Qiu X., Sun T., Xu Y., Shao Y., Dai N., Huang X. (2020). Pre-trained models for natural language processing: A survey. Sci. China Technol. Sci..

[16] Liu Y., Zhang Y., Wang Y., Hou F., Yuan J., Tian J., Zhang Y., Shi Z., Fan J., He Z. (2021). A survey of visual transformers. arXiv.

[17] Phan H., McLoughlin I.V., Pham L., Chén O.Y., Koch P., De Vos M., Mertins A. (2020). Improving GANs for speech enhancement. IEEE Signal Process. Lett..

[18] Pascual S., Bonafonte A., Serra J. (2017). SEGAN: Speech enhancement generative adversarial network. arXiv.

[19] Fu S.W., Liao C.F., Tsao Y., Lin S.D. Metricgan: Generative adversarial networks based black-box metric scores optimization for speech enhancement. Proceedings of the International Conference on Machine Learning, PMLR.

[20] Soni M.H., Shah N., Patil H.A. Time-frequency masking-based speech enhancement using generative adversarial network. Proceedings of the 2018 IEEE International Conference on Acoustics, Speech and Signal Processing (ICASSP).

[21] Donahue C., Li B., Prabhavalkar R. Exploring speech enhancement with generative adversarial networks for robust speech recognition. Proceedings of the 2018 IEEE International Conference on Acoustics, Speech and Signal Processing (ICASSP).

[22] Wang D. (2005). On ideal binary mask as the computational goal of auditory scene analysis. Speech Separation by Humans and Machines.

[23] Narayanan A., Wang D. Ideal ratio mask estimation using deep neural networks for robust speech recognition. Proceedings of the 2013 IEEE International Conference on Acoustics, Speech and Signal Processing.

[24] Williamson D.S., Wang Y., Wang D. (2015). Complex ratio masking for monaural speech separation. IEEE/ACM Trans. Audio Speech Lang. Process..

[25] Heymann J., Drude L., Haeb-Umbach R. Neural network based spectral mask estimation for acoustic beamforming. Proceedings of the 2016 IEEE International Conference on Acoustics, Speech and Signal Processing (ICASSP).

[26] Tu Y.H., Du J., Lee C.H. DNN Training Based on Classic Gain Function for Single-channel Speech Enhancement and Recognition. Proceedings of the ICASSP 2019—2019 IEEE International Conference on Acoustics, Speech and Signal Processing (ICASSP).

[27] Strake M., Defraene B., Fluyt K., Tirry W., Fingscheidt T. Separated Noise Suppression and Speech Restoration: Lstm-Based Speech Enhancement in Two Stages. Proceedings of the 2019 IEEE Workshop on Applications of Signal Processing to Audio and Acoustics (WASPAA).

[28] Ali M.N., Brutti A., Falavigna D. Speech enhancement using dilated wave-u-net: An experimental analysis. Proceedings of the 2020 IEEE 27th Conference of Open Innovations Association (FRUCT).

[29] Yang M., Ma M.Q., Li D., Tsai Y.H.H., Salakhutdinov R. Complex transformer: A framework for modeling complex-valued sequence. Proceedings of the ICASSP 2020—2020 IEEE International Conference on Acoustics, Speech and Signal Processing (ICASSP).

[30] Kim J., El-Khamy M., Lee J. T-gsa: Transformer with gaussian-weighted self-attention for speech enhancement. Proceedings of the ICASSP 2020—2020 IEEE International Conference on Acoustics, Speech and Signal Processing (ICASSP).

[31] Yu W., Zhou J., Wang H., Tao L. (2022). SETransformer: Speech enhancement transformer. Cogn. Comput..

[32] Zaheer M., Guruganesh G., Dubey K.A., Ainslie J., Alberti C., Ontanon S., Pham P., Ravula A., Wang Q., Yang L. (2020). Big bird: Transformers for longer sequences. Adv. Neural Inf. Process. Syst..

[33] Zeng A., Chen M., Zhang L., Xu Q. (2022). Are Transformers Effective for Time Series Forecasting?. arXiv.

[34] Hu J., Shen L., Sun G. Squeeze-and-excitation networks. Proceedings of the IEEE Conference on Computer Vision and Pattern Recognition.

[35] Snyder D., Chen G., Povey D. (2015). Musan: A music, speech, and noise corpus. arXiv.

[36] Veaux C., Yamagishi J., MacDonald K. (2017). CSTR VCTK Corpus: English Multi-Speaker Corpus for CSTR Voice Cloning Toolkit. https://www.semanticscholar.org/paper/SUPERSEDED-CSTR-VCTK-Corpus%3A-English-Multi-speaker-Veaux-Yamagishi/d4903c15a7aba8e2c2386b2fe95edf0905144d6a.

[37] Varga A., Steeneken H.J. (1993). Assessment for automatic speech recognition: II. NOISEX-92: A database and an experiment to study the effect of additive noise on speech recognition systems. Speech Commun..

[38] Pigeon D.I.S. (2022). My Noise. https://mynoise.net/NoiseMachines/cafeRestaurantNoiseGenerator.php.

[39] Loshchilov I., Hutter F. Fixing Weight Decay Regularization in Adam. Proceedings of the 6th International Conference on Learning Representations, ICLR 2018.

[40] Tan K., Wang D. A Convolutional Recurrent Neural Network for Real-Time Speech Enhancement. Proceedings of the Interspeech.

